# Maternal selenium status in pregnancy, offspring glutathione peroxidase 4 genotype, and childhood asthma

**DOI:** 10.1016/j.jaci.2014.10.035

**Published:** 2015-04

**Authors:** Seif O. Shaheen, Clare M. Rutterford, Sarah J. Lewis, Susan M. Ring, John W. Holloway, Jean Golding, A. John Henderson

**Affiliations:** a Centre for Primary Care and Public Health, Barts and The London School of Medicine and Dentistry, London, United Kingdom; b School of Social and Community Medicine, University of Bristol, Bristol, United Kingdom; c MRC Integrative Epidemiology Unit at the University of Bristol, Bristol, United Kingdom; d Human Development and Health, Faculty of Medicine, University of Southampton, Southampton, United Kingdom

To the Editor:

Two prospective birth cohort studies have suggested that prenatal selenium status may play a role in the inception of childhood asthma. In the Avon Longitudinal Study of Parents and Children (ALSPAC), umbilical cord tissue concentration of selenium was negatively associated with risk of wheezing in early childhood^1^; in another United Kingdom cohort, maternal and cord plasma selenium concentrations were negatively associated with wheezing in the second year of life, but not at 5 years.^2^ Associations between biomarkers of prenatal selenium exposure and asthma and wheezing later in childhood have not been reported.

Glutathione peroxidase (GPX) 4 is a unique membrane-associated selenium-dependent antioxidant enzyme that can directly reduce phospholipid hydroperoxides and protect against oxidative stress in mammalian cells.^3^ Aside from its antioxidant role, *GPX4* has been implicated in lipoxygenase metabolism,^4^ which has major relevance to leukotriene synthesis and inflammatory signaling in asthma. Thus, demonstration of an interaction between selenium status and *GPX4* genotype on asthma risk could strengthen the evidence for a causal role of selenium.

In the ALSPAC birth cohort, we investigated whether higher maternal blood concentration of selenium in pregnancy is associated with a lower risk of asthma and wheezing at age 7 years and whether associations are modified by the *GPX4* genotype (rs713041). Details of the ALSPAC protocol can be found at http://www.alspac.bris.ac.uk. Methods for maternal selenium analysis have been described in detail previously^5^; whole blood samples were obtained from 4484 women as early as possible in pregnancy, and selenium analysis was performed by the Centers for Disease Control and Prevention, Atlanta, Georgia, in 2009-2010. After excluding assay failures, selenium measurements were complete for 4287 women. The mean (interquartile range) of the gestational timing of blood samples was 11.7 (9-13) weeks (median, 11 weeks; range, 2-42 weeks). Maternal blood selenium concentrations were log transformed for analysis. Children were defined as having current doctor-diagnosed asthma at 7.5 years if mothers responded positively to the question “Has a doctor *ever* actually *said* that your study child has asthma?” *and* positively to questions about wheezing and/or asthma in the past 12 months at 7.5 years. A single nucleotide polymorphism (SNP) in *GPX4* (rs713041, at position 718) was typed by LGC Genomics Ltd (Hoddesdon, Herts, United Kingdom; www.lgcgenomics.com) in mothers and children using a competitive allele-specific PCR system (KASPar). Maternal and child *GPX4* genotype frequencies did not deviate from Hardy-Weinberg equilibrium. All analyses were restricted to white mothers and their offspring. The median blood selenium concentration was 110.37 μg/L (range, 27.4-324.07 μg/L); the arithmetic mean was 113.82 ± 24.53 μg/L. The 20% centile concentration was 96 μg/L, which approximates to the whole blood concentration of 100 μg/L needed to saturate glutathione peroxidase activity in adults *in vivo.*^6^ After controlling for confounders, including gestational timing of maternal blood samples (see this article's Online Repository at www.jacionline.org for the full list), it was found that maternal blood selenium concentration was not associated with childhood asthma (N = 2298) or wheezing (N = 2326) overall (see Table E1 in this article's Online Repository at www.jacionline.org), nor was maternal or child *GPX4* genotype associated with these outcomes (see Table E2 in this article's Online Repository at www.jacionline.org). Table I presents the adjusted associations between maternal blood selenium concentrations and asthma and wheezing, stratified by maternal and child *GPX4* genotypes. Maternal genotype did not modify the associations. However, there was evidence of interaction between maternal selenium status and child *GPX4* genotype on asthma (*P* interaction .068) and wheezing (*P* interaction .011), with an odds reduction of 83% to 84% per doubling increase in blood selenium concentration in children who were homozygous for the minor T allele of rs713041; maternal blood selenium concentration was not associated with these outcomes in children carrying the C allele. Similarly, children of mothers below the 20th centile for blood selenium concentration were more likely to have asthma than were children of mothers with higher blood selenium concentration if they were homozygous for the T allele of rs713041 (odds ratio, 2.21; 95% CI, 0.98-4.99; *P* = .057), but not if they were carrying the C allele (*P* interaction .18) (Fig 1). The main findings were unchanged when we carried out 2 sensitivity analyses: first, excluding 3 mother-child pairs with outlying maternal blood selenium values (>300 μg/L); second, after controlling for 10 variables derived by principal-components analysis from the ALSPAC genomewide association study data to address possible residual confounding by population substructure.

In this population-based birth cohort study, we have found a novel, and plausible, interaction between maternal blood selenium concentration in pregnancy and child *GPX4* genotype (rs713041) on the risk of asthma and wheezing at age 7 years. Our data indicate that low maternal blood selenium concentration increases the risk and higher blood selenium concentration reduces the risk but only in genetically susceptible children, namely, those who are homozygous for the minor T allele of *GPX4*. Villette et al^4^ proposed that the rs713041 SNP in the 3′ untranslated region of *GPX4* may influence how efficiently selenocysteine is incorporated into *GPX4*, leading to altered *GPX4* synthesis in response to variations in selenium supply.^4^ There is other evidence confirming that T/C variation (rs713041) has functional consequences,^7^ including a study showing that in individuals with the *GPX4* (rs713041) TT genotype, oxidative stress decreased as *in vivo* selenium concentrations Furthermore, *in vitro*, selenium is necessary for the maximum expression of *GPX4* in human lung epithelial cells.^9^ We therefore propose that low maternal blood selenium concentration leads to suboptimal *GPX4* activity and impaired antioxidant defenses against oxidative stress in fetal airway epithelium, leading to epithelial damage, which, in turn, contributes to the pathogenesis of asthma. Alternatively, prenatal selenium status might influence the development of asthma through the involvement of *GPX4* in lipoxygenase metabolism.^4^ However, because we cannot exclude the possibility that the main findings have arisen through type 1 error, replication of the interaction we observed is needed in another birth cohort of sufficient size.

Our findings suggest that in a susceptible subgroup of mother-child pairs in the population, namely, pregnant women with suboptimal selenium status and offspring with the homozygous minor variant of *GPX4* rs71304, there may be potential to reduce the incidence of childhood asthma through selenium supplementation in pregnancy. Whether primary prevention of asthma can be achieved in this way can be determined only through a clinical trial; the efficacy of such a trial is likely to be maximized by adopting a stratified approach.

## Figures and Tables

**Table E1:** Associations between maternal blood selenium concentrations during pregnancy and childhood asthma and wheezing

Disease	Selenium concentration (μg/L)	Unadjusted OR (95% CI)	Adjusted OR (95% CI)
Asthma (N = 2298)	Q1 (≤96)	1.0	1.0
	Q2 (96-105.74)	0.77 (0.52-1.14)	0.82 (0.54-1.26)
	Q3 (105.8-115.47)	0.61 (0.40-0.92)	0.76 (0.49-1.18)
	Q4 (115.5-128.7)	0.73 (0.49-1.08)	0.85 (0.55-1.31)
	Q5 (128.8-324.07)	0.72 (0.48-1.07)	0.83 (0.53-1.30)
	Per doubling	0.66 (0.42-1.04)	0.74 (0.45-1.21)
		*P* value for trend = .07	*P* value for trend = .24
Wheezing (N = 2326)	Q1 (≤96)	1.0	1.0
	Q2 (96-105.74)	0.92 (0.60-1.40)	0.97 (0.61-1.54)
	Q3 (105.8-115.47)	0.77 (0.50-1.20)	0.96 (0.59-1.54)
	Q4 (115.5-128.7)	1.05 (0.70-1.59)	1.16 (0.73-1.84)
	Q5 (128.8-324.07)	0.95 (0.62-1.45)	1.07 (0.66-1.72)
	Per doubling	0.84 (0.52-1.33)	0.87 (0.52-1.45)
		*P* value for trend = .45	*P* value for trend = .59

*OR*, Odds ratio.

**Table E2:** Association between maternal and child *GPX4* rs713041 genotype and childhood asthma and wheezing

Disease	Maternal *GPX4* rs713041 genotype		OR (95% CI)
Asthma (N = 1738)	N		
	560	C:C	1.0
	852	T:C	0.90 (0.64-1.25)
	326	T:T	1.29 (0.87-1.93)
		Per allele	1.11 (0.91-1.37)
Wheezing (N = 1761)	N		
	567	C:C	1.0
	865	T:C	0.83 (0.59-1.19)
	329	T:T	1.28 (0.85-1.94)
		Per allele	1.10 (0.89-1.37)

Disease	Child *GPX4* rs713041 genotype		OR (95% CI)
Asthma (N = 1748)	N		
	573	C:C	1.0
	877	T:C	1.23 (0.88-1.73)
	298	T:T	1.24 (0.80-1.94)
		Per allele	1.13 (0.91-1.40)
Wheezing (N = 1772)	N		
	577	C:C	1.0
	893	T:C	0.88 (0.63-1.24)
	302	T:T	1.10 (0.71-1.71)
		Per allele	1.02 (0.82-1.28)

*OR*, Odds ratio.

**Fig 1:**
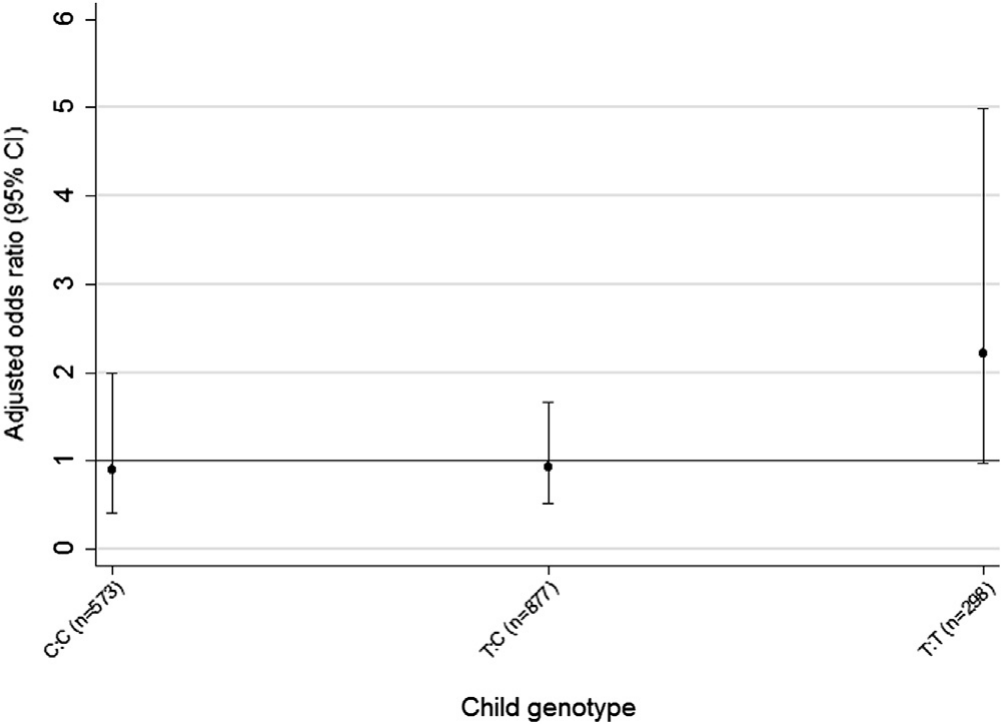
Odds ratio for asthma, comparing children of mothers in the bottom quintile for blood selenium concentration with children of mothers in the top 4 quintiles, and stratifying by child *GPX4* rs713041 genotype.

**Table I:** Associations between maternal blood selenium concentrations during pregnancy and childhood asthma and wheezing, stratified by maternal and child *GPX4* rs713041 genotype

*GPX4* rs713041 genotype	N	Adjusted OR (95% CI) per doubling selenium concentration	*P* value	N	Adjusted OR (95% CI) per doubling selenium concentration	*P* value
Asthma	Stratified by maternal genotype			Stratified by child genotype		
C:C	560	0.51 (0.17-1.54)	.23	573	0.92 (0.31-2.74)	.88
T:C	852	0.95 (0.41-2.22)	.91	877	1.18 (0.50-2.78)	.70
T:T	326	0.92 (0.29-2.92)	.88	298	0.17 (0.04-0.72)	.02
*P* for interaction∗	.64			.068		
Wheezing	Stratified by maternal genotype			Stratified by child genotype		
C:C	567	0.74 (0.24-2.27)	.60	577	0.69 (0.26-1.86)	.46
T:C	865	1.41 (0.57-3.45)	.46	893	1.99 (0.81-4.93)	.13
T:T	329	1.14 (0.35-3.66)	.83	302	0.16 (0.04-0.66)	.01
*P* for interaction∗	.68			.011		

*OR*, Odds ratio.

∗ Testing difference in selenium associations according to genotype.

## Confounders

In multivariate analyses, we controlled for the following potential confounders: maternal factors during pregnancy (smoking, infections, anxiety score, and use of antibiotics, alcohol, and paracetamol); other maternal factors (educational level, housing tenure, financial difficulties, body mass index, age, parity, and history of asthma, eczema, rhinoconjunctivitis, and migraine); sex of child, season of birth, multiple pregnancy, gestational age, birth weight, head circumference, and birth length; and postnatal factors (breast-feeding, day care, pets, damp/mold, environmental tobacco smoke exposure, antibiotic and paracetamol use in infancy, number of younger siblings, and body mass index at age 7 years). We also controlled for the gestational timing of maternal blood samples because whole blood selenium concentrations are known to decline as pregnancy progresses.^E1^

## Statistical analysis

To test for interaction, we included the genotype and selenium variables, as well as a Genotype × Selenium interaction term, in the regression models.
